# Supplementary material for: Hybrid Passivated Red Organic LEDs with Prolonged Operation and Storage Lifetime
Source: Molecules. 2022 Apr 19;27(9):2607. doi: 10.3390/molecules27092607 (PMC9099473; doi:10.3390/molecules27092607)
Supplement: Supplementary file 1 [file molecules-27-02607-s001.zip › molecules-1664365-supplementary.pdf]

Supplementary Materials

Hybrid Passivated Red Organic LEDs with Prolonged Operation and Storage Lifetime

Dan-Dan Feng [1], Shuang-Qiao Sun [1], Wei He [1], Jun Wang [2], Xiao-Bo Shi [2] and Man-Keung Fung [1,2,3,*]

[1] Institute of Functional Nano & Soft Materials (FUNSOM), Jiangsu Key Laboratory for Carbon-Based Functional Materials & Devices, Soochow University, Suzhou 215123, China; change071125@163.com (D.-D.F.); 20214014016@stu.suda.edu.cn (S.-Q.S.); 20204014026@stu.suda.edu.cn (W.H.)

[2] Institute of Organic Optoelectronics, Jiangsu Industrial Technology Research Institute (JITRI), Wujiang, Suzhou 215123, China; wangj@jitriioo.com (J.W.); shixb@jitriioo.com (X.-B.S.)

[3] Macao Institute of Materials Science and Engineering (MIMSE), MUST-SUDA Joint Research Center for Advanced Functional Materials, Zhuhai MUST Science and Technology Research Institute, Macau University of Science and Technology, Macau 999078, China

* Correspondence: mkfung@suda.edu.cn

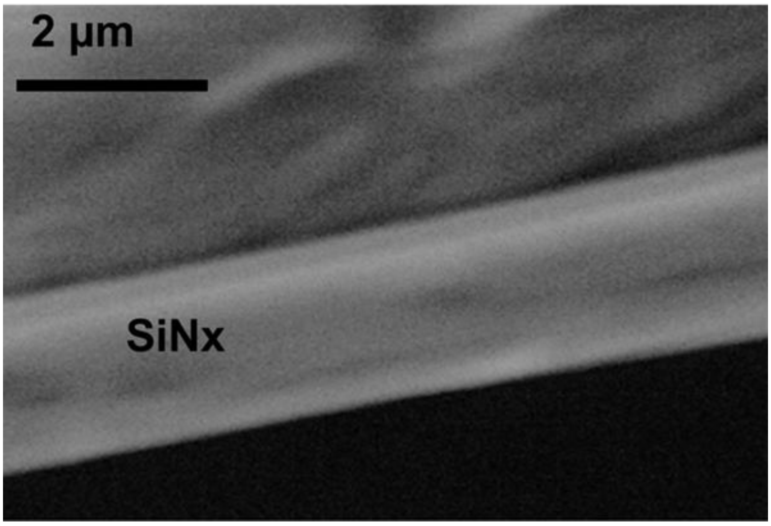

Figure S1. Cross-sectional SEM image of a 2 μm thick Si_xN_y encapsulation film.

Table S1. Residual stress of Si_xN_y and Si_xN_y/Al measured in different film preparation conditions.

Flim Type		Stress(MPa)						
Si_xN_y	Deposition Temperature(°C)	55	65	75	80	85	90	95
		-178.3	-88.5	-47.3	-35.0	-23.2	-28.4	-30.1
	Thickness(μm)	0.5	1	1.5	2	2.5		
		-25.7	-12.6	-19.7	-23.2	-46.4		
Al	Thickness(nm)	100	200	300	400	500		
		5.4	10.3	21.1	45.1	57.2		
	Si_xN_y/Al	2 μm/300 nm						
		-1.0						

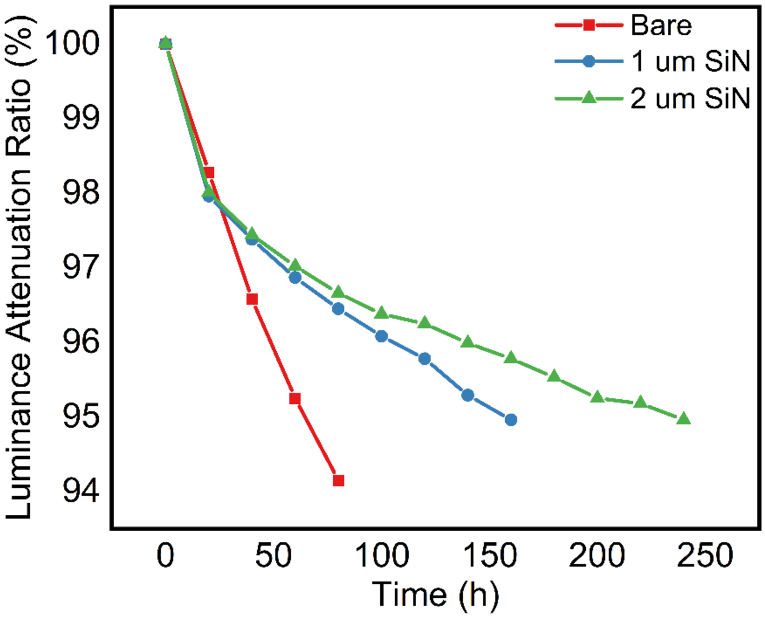

Figure S2. Operation lifetime of devices without any encapsulation and encapsulated with 1um and 2um thick Si_xN_y. All devices were driven at a current density of 50 mA/cm^2.

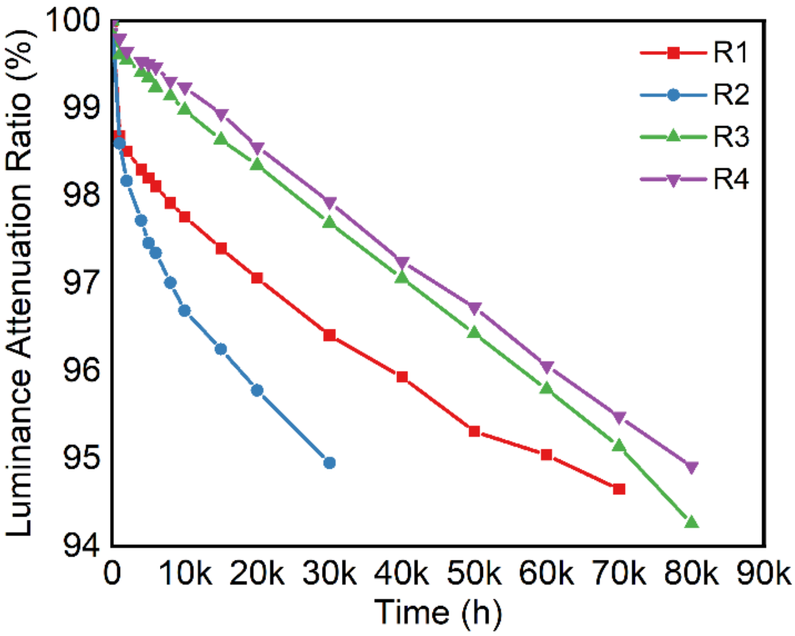

Figure S3. T_{95} operation lifetime at a luminance of 1000 cd/m^2.

Table S2. Luminous surface of a bare device without encapsulation and a device solely encapsulated with a glass cover under the 85/85 test.

Type	Fresh	5 h	20 h	50 h
Bare	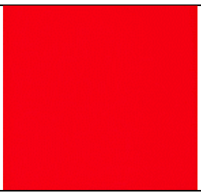	---		
Glass cover	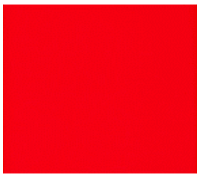	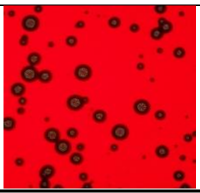	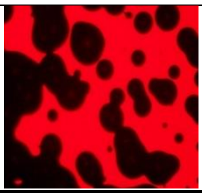	---
